# Supplementary material for: Two Modified Questionnaires for the Assessment of Nutrition Impact Symptoms in the Rehabilitation Phase after Burn Injury: A Content Validation Study
Source: Eur Burn J. 2022 Feb 18;3(1):156–64. doi: 10.3390/ebj3010013 (PMC11575375; doi:10.3390/ebj3010013)
Supplement: Supplementary file 1 [file ebj-03-00013-s001.zip › ebj-1519899-supplementary.pdf]

Supplementary Materials File S1

Disease Related Appetite Questionnaire modified version, DRAQ-burn

This questionnaire has statements and questions about your appetite and food habits. The aim is to get an understanding of which symptoms that may influence your appetite and food habits at present.

Read every statement and check the box for the alternative that best fits your present situation. If you are not sure, check the box that feels most appropriate for you.

1. **My appetite is…**
   - ☐ very poor
   - ☐ poor
   - ☐ neither poor nor good
   - ☐ good
   - ☐ very good

2. **My appetite varies from day to day – some days its good, other days its poor**
   - ☐ Fully agree
   - ☐ Partially agree
   - ☐ Neither agree nor disagree
   - ☐ Partially disagree
   - ☐ Fully disagree

3. **When I eat…**
   - ☐ I feel full after eating a couple of bites of the meal
   - ☐ I feel full after eating ¼ of the meal
   - ☐ I feel full after eating approximately half of the meal
   - ☐ I feel full after eating all or almost all of the meal
   - ☐ I usually don´t feel full

4. **I feel hungry…**
   - ☐ Never
   - ☐ Rarely
   - ☐ Sometimes
   - ☐ Often
   - ☐ Always

5. **Compared to before injury, food tastes…**
   - ☐ A lot worse
   - ☐ Worse
   - ☐ Neither worse nor better
   - ☐ Better
   - ☐ A lot better

6. I feel nauseated when I eat

- ☐ Always
- ☐ Often
- ☐ Sometimes
- ☐ Rarely
- ☐ Never

7. How often do you eat anything?

- ☐ 0-2 times a day
- ☐ 3-4 times a day
- ☐ 5-6 times a day
- ☐ 7-8 times a day
- ☐ more than 8 times a day

8. My eating varies from day to day – some days I eat more, other days less

- ☐ Fully agree
- ☐ Partially agree
- ☐ Neither agree nor disagree
- ☐ Partially disagree
- ☐ Fully disagree

9. For how long have your injury affected your appetite?

- ☐ Not at all
- ☐ Less than 2 weeks
- ☐ 2 weeks – 3 months
- ☐ 3-6 months
- ☐ More than 6 months

10. I use oral nutritional supplements (prescribed from health-care provider)

- ☐ Yes
- ☐ No

11. Because of my burn injury I depend on someone else to prepare my meals

- ☐ Always
- ☐ Often
- ☐ Sometimes
- ☐ Rarely
- ☐ Never

12. My dressings, scars, or itching <u>affect my appetite</u>

- ☐ Fully agree
- ☐ Partially agree
- ☐ Neither agree nor disagree
- ☐ Partially disagree
- ☐ Fully disagree

13. My dressings, scars, or itching <u>prevents me from eating</u>

- ☐ Fully agree
- ☐ Partially agree
- ☐ Neither agree nor disagree
- ☐ Partially disagree
- ☐ Fully disagree

14. My reduced functional ability <u>prevents me from eating</u>

- ☐ Fully agree
- ☐ Partially agree
- ☐ Neither agree nor disagree
- ☐ Partially disagree
- ☐ Fully disagree

Supplementary Materials File S2

Eating Symptom Questionnaire modified version, ESQ-burn

During the last 2 weeks have you had the following symptoms? Check the box that is most accurate for you.

		No symptoms	Mild symptoms	Moderate symptoms	Moderately severe symptoms	Severe symptoms
1.	Nausea	☐	☐	☐	☐	☐
2.	Pain or ache affecting my appetite	☐	☐	☐	☐	☐
3.	Difficulties chewing	☐	☐	☐	☐	☐
4.	Difficulties swallowing	☐	☐	☐	☐	☐
5.	Pain or ache preventing me from eating	☐	☐	☐	☐	☐
6.	Tiredness, low energy (fatigue) affecting my appetite	☐	☐	☐	☐	☐
7.	Tiredness, low energy (fatigue) preventing me from eating	☐	☐	☐	☐	☐

8. Do you have *other symptoms* that affect your appetite or prevent you from eating?
